# Is early post-operative treatment with 5-fluorouracil possible without affecting anastomotic strength in the intestine?

**DOI:** 10.1038/sj.bjc.6690086

**Published:** 1999-02

**Authors:** B M van der Kolk, B M de Man, T Wobbes, T Hendriks

**Affiliations:** Department of Surgery, University Hospital Nijmegen, Nijmegen, The Netherlands

**Keywords:** anastomosis, fluorouracil, healing, intestine, rat, strength

## Abstract

Early post-operative local or systemic administration of 5-fluorouracil (5-FU) is under investigation as a means to improve outcome after resection of intestinal malignancies. It is therefore quite important to delineate accurately its potentially negative effects on anastomotic repair. Five groups (*n* = 24) of rats underwent resection and anastomosis of both ileum and colon: a control group and four experimental groups receiving daily 5-FU, starting immediately after operation or after 1, 2 or 3 days. Within each group, the drug (or saline) was delivered either intraperitoneally (*n* = 12) or intravenously (*n* = 12). Animals were killed 7 days after operation and healing was assessed by measurement of anastomotic bursting pressure, breaking strength and hydroxyproline content. In all cases, 5-FU treatment from the day of operation or from day 1 significantly (*P* < 0.025) and severely suppressed wound strength; concomitantly, the anastomotic hydroxyproline content was reduced. Depending on the location of the anastomosis and the route of 5-FU administration, even a period of 3 days between operation and first dosage seemed insufficient to prevent weakening of the anastomosis. The effects of intravenous administration, though qualitatively similar, were quantitatively less dramatic than those observed after intraperitoneal delivery. Post-operative treatment with 5-FU, if started within the first 3 days after operation, is detrimental to anastomotic strength and may compromise anastomotic integrity. © 1999 Cancer Research Campaign


*British Journal of Cancer* (1999) **79**(3/4), 545–550
© 1999 Cancer Research Campaign
Article no. bjoc.1998.0086

### Is early post-operative treatment with 5-fluorouracil possible without affecting anastomotic strength in the intestine?

**BM van der Kolk, BM de Man, T Wobbes and T Hendriks**

Department of Surgery, University Hospital Nijmegen, Nijmegen, The Netherlands

**Summary** Early post-operative local or systemic administration of 5-fluorouracil (5-FU) is under investigation as a means to improve outcome after resection of intestinal malignancies. It is therefore quite important to delineate accurately its potentially negative effects on anastomotic repair. Five groups ($n$ = 24) of rats underwent resection and anastomosis of both ileum and colon: a control group and four experimental groups receiving daily 5-FU, starting immediately after operation or after 1, 2 or 3 days. Within each group, the drug (or saline) was delivered either intraperitoneally ($n$ = 12) or intravenously ($n$ = 12). Animals were killed 7 days after operation and healing was assessed by measurement of anastomotic bursting pressure, breaking strength and hydroxyproline content. In all cases, 5-FU treatment from the day of operation or from day 1 significantly ($P < 0.025$) and severely suppressed wound strength; concomitantly, the anastomotic hydroxyproline content was reduced. Depending on the location of the anastomosis and the route of 5-FU administration, even a period of 3 days between operation and first dosage seemed insufficient to prevent weakening of the anastomosis. The effects of intravenous administration, though qualitatively similar, were quantitatively less dramatic than those observed after intraperitoneal delivery. Post-operative treatment with 5-FU, if started within the first 3 days after operation, is detrimental to anastomotic strength and may compromise anastomotic integrity.

**Keywords:** anastomosis; fluorouracil; healing; intestine; rat; strength

In the majority of patients with colorectal carcinoma the tumour will be resectable, and thus potentially curable, at the time of presentation. However, despite the high resectability rate and improving surgical therapy, nearly half of the patients will ultimately die from recurrent disease. Safe and effective adjuvant therapy is desperately needed, particularly for patients with Dukes' stage $B_{2–3}$ and C carcinoma, which constitute 60–70% of the population presenting with colorectal cancer.

Since its introduction over 35 years ago, 5-fluorouracil (5-FU) has remained the cornerstone of adjuvant treatment of colorectal carcinoma. However, none of the regimens currently available is a satisfactory answer to the problem of recurrent disease. Efforts to devise more effective adjuvant treatments include various approaches. In addition to attempts to modulate biochemically the cytotoxic activity of 5-FU, these include the development of new agents and of immunotherapy (Fuchs and Mayer, 1995; Sinicrope and Sugarman, 1995; Rustum et al, 1997). Another approach comprises the investigation of local–regional treatment strategies, in particular administration of drugs into the portal vein or intraperitoneally (Saltz and Kelsen, 1997). It has been suggested that the beneficial effects of portal vein infusion are attributable to the effects of immediate, perioperative systemic treatment rather than to its hepatic-directed nature (Vaughn and Haller, 1997). Also, early use of intraperitoneal treatment may be needed for best results (Cunliffe and Sugarbaker, 1989; Saltz and Kelsen, 1997).

*Received 11 March 1998*
*Revised 2 May 1998*
*Accepted 4 June 1998*

*Correspondence to:* T Hendriks, Department of Surgery, University Hospital Nijmegen, PO Box 9101, 6500 HB Nijmegen, The Netherlands

The apparent interest in immediate post-operative local and/or systemic adjuvant 5-FU therapy necessitates the delineation of its hazards for anastomotic repair in the intestine. The early phase of anastomotic healing is characterized by a transient loss of strength in the anastomosed segment (Hendriks and Mastboom, 1990). Further reduction in wound strength in this period may compromise anastomotic integrity and increase the risk of anastomotic dehiscence, which is a potentially devastating surgical complication. Previous studies in our laboratory have shown that administration of 5-FU on the day of surgery and on the next 2 days does not significantly reduce strength in experimental intestinal anastomoses (de Waard et al, 1993, 1995*a*). However, a 7-day course of intraperitoneal 5-FU, starting on the day of surgery, severely reduces anastomotic strength as measured 7 days after operation (Graf et al, 1992; de Waard et al, 1995*b*). The present study addresses the question of whether postponement of the start of 5-FU administration until the first, second or third day after operation can prevent these negative effects on the development of anastomotic strength during the first post-operative week.

### MATERIALS AND METHODS

### Animals

Altogether, 120 male outbred Wistar/Cpb:WU rats, weighing between 250 and 320 g, were used. They were housed two animals per cage and had free access to water and standard laboratory chow (diet AM II, Hope Farms, Woerden, The Netherlands). The animals were divided into two equal groups, one for the experiment with intravenous 5-FU, the other for the experiment with intraperitoneal 5-FU. Within each group, five subgroups of 12 rats each were formed: a control group (C) and four experimental

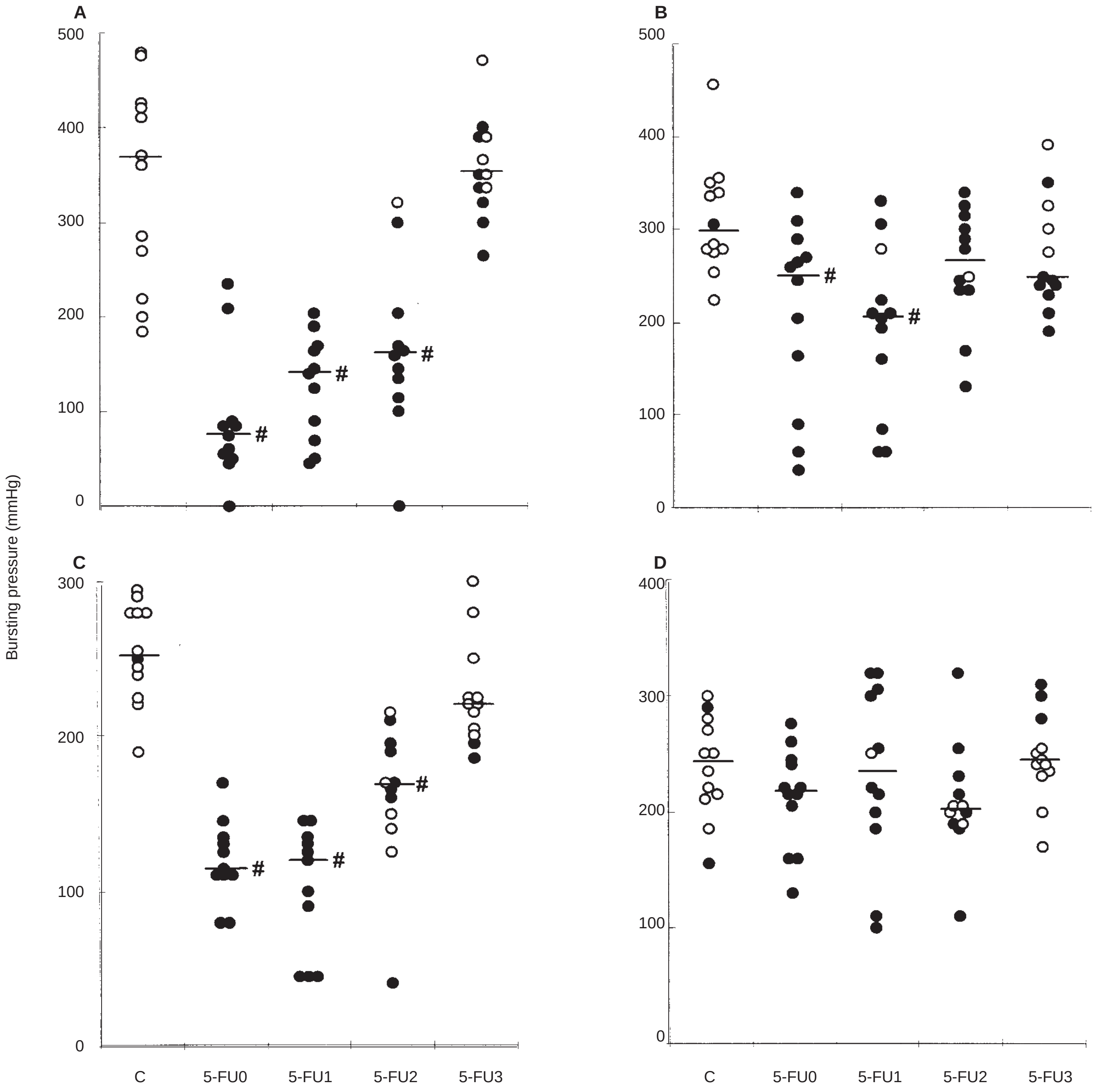

**Figure 1** Bursting pressure in anastomotic segments. Circles represent individual animals and horizontal bars the median values; open circles, rupture outside anastomosis; closed circles, rupture within suture line. (**A**) Ileum, intraperitoneal 5-FU. (**B**) Ileum, intravenous 5-FU. (**C**) Colon, intraperitoneal 5-FU. (**D**) Colon, intravenous 5-FU. #Significant ($P < 0.025$, see Materials and methods) difference between experimental groups and the control group groups, which received 5-FU starting on the day of surgery (5-FU0) or on the first (5-FU1), second (5-FU2) or third (5-FU3) post-operative day. All animals were killed 7 days after operation.

The study was approved by the Animal Ethics Review Committee of the Faculty of Medicine, University of Nijmegen.

### Drug administration

5-FU (Abic, Netanya, Israel) was given in a dose of 20 mg $kg^{-1}$ body weight once a day. This is the same dose that we used previously (de Waard et al, 1993, 1995*a*,*b*) and represents the highest dose which, in combination with surgery, did not result in a significant mortality. 5-FU was injected as a bolus in a concentration of 1 mg $ml^{-1}$ saline intraperitoneally and in a concentration of 2 mg $ml^{-1}$ saline intravenously (through the vein of the tail). In those experimental groups in which 5-FU was not started immediately after operation, animals received daily saline instead. The animals in the two control groups received saline daily throughout the experiment.

### Operative procedure

During halothane anaesthesia, a midline incision was made and 1 cm of both small and large bowel were resected, at 15 cm proximal to the ileocaecal junction, and 3 cm proximal to the rectal peritoneal

*© Cancer Research Campaign 1999*

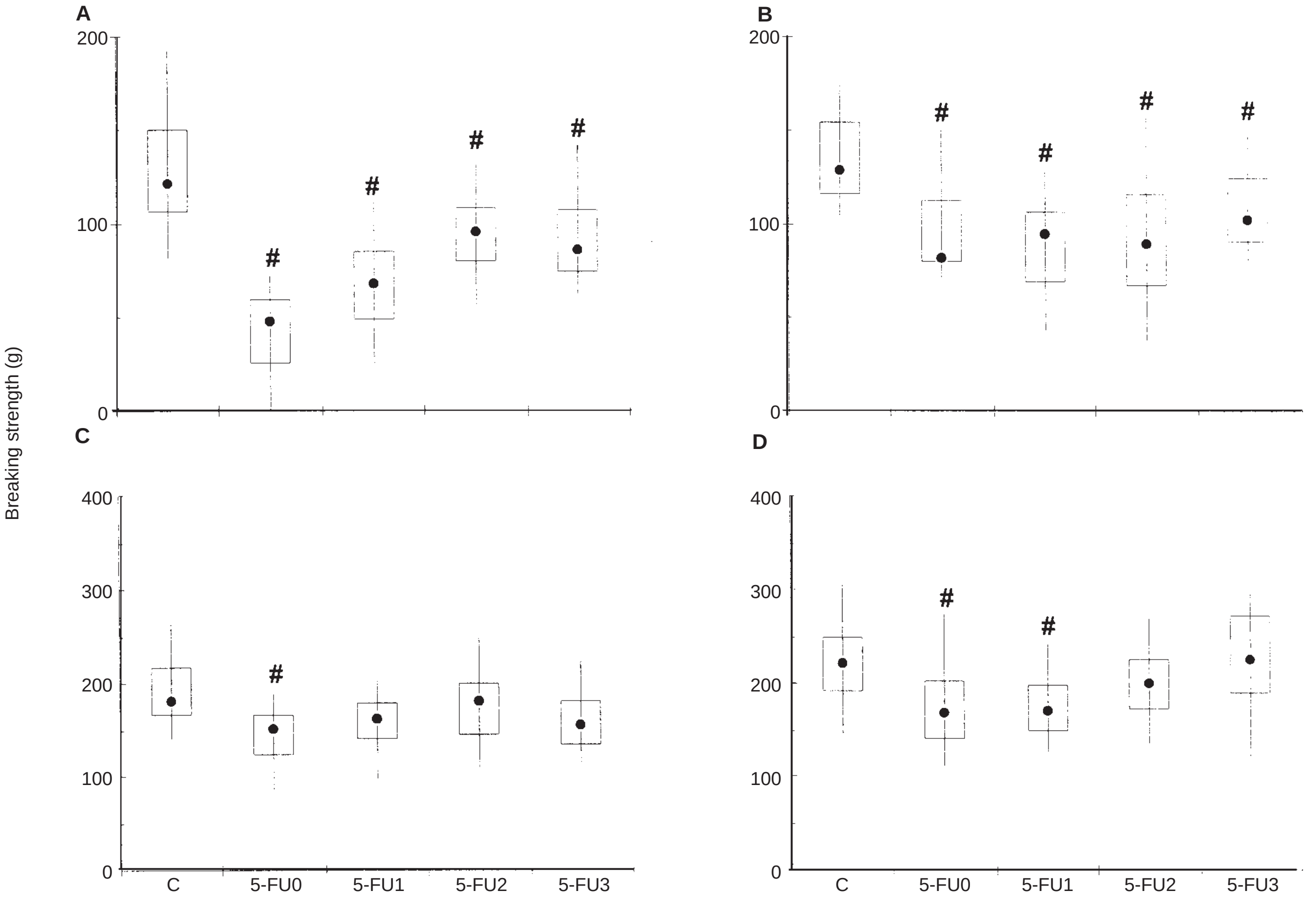

**Figure 2** Anastomotic breaking strength. Data represent median values (●) and range (vertical lines); boxes delineate the 95% confidence interval. (**A**) Ileum, intraperitoneal 5-FU. (**B**) Ileum, intravenous 5-FU. (**C**) Colon, intraperitoneal 5-FU. (**D**) Colon, intravenous 5-FU. #Significant (*P* < 0.025, see Materials and methods) difference between experimental groups and the control group reflection respectively. Continuity was restored microsurgically by the construction of an inverted one-layer seromuscular end-to-end anastomosis with eight interrupted sutures of 8 × 0 monofilament material (Ethicon, Sommerville, NJ, USA). The abdomen was closed in two layers with a continuous 3 × 0 silk suture for the fascia and staples for the skin.

### Analytical procedures

The rats were killed by an intraperitoneal overdose of sodium pentobarbital. After opening the abdominal wound and identifying the anastomoses, the adhesions were cut as far as possible without injuring the intestine. An intestinal segment with the anastomosis in the middle was removed, with the sutures left in place. This segment was attached to an infusion pump filled with methylene blue-stained saline. The pressure was raised with an infusion rate of 4 ml $min^{-1}$ and recorded graphically. Both the bursting pressure, that is the maximum pressure recorded immediately before sudden loss of pressure, and the site of rupture were noted. Thereafter, the segment was placed in a tensiometer and the breaking strength was recorded. Thus, both the bursting pressure and breaking strength were measured in the same anastomotic segment. The validity of this procedure had been confirmed in a pilot experiment. Anastomotic breaking strength was compared in two groups of rats, either measured directly or after the procedure used for measuring the bursting pressure, and found to be similar in both groups (de Waard et al, 1995*a*). The anastomotic segment was then cleaned from the surrounding tissue and a 5-mm segment with the suture line in the middle was collected. The samples were frozen immediately and stored in liquid nitrogen until processing. After weighing, the samples were pulverized and lyophilized and the hydroxyproline content was measured by high-performance liquid chromatography (HPLC) after hydrolysis with 6 N hydrochloric acid and derivatization with dabsyl chloride.

### Statistical analysis

To correct for the fact that multiple comparisons were made, pair-wise comparisons of groups were performed (with a two-tailed Mann–Whitney test) using a level of significance of $\alpha' = 2\,\alpha/k$, where $k$ is the number of pairwise comparisons. For instance, differences between the four experimental groups and the control group were considered significant ($\alpha = 0.05$) at $P < \alpha'$, where $\alpha' = 2 \times 0.05/4 = 0.025$.

### RESULTS

During the experiment two animals died prematurely, both from groups receiving intraperitoneal 5-FU. In the 5-FU0 group, one rat died from anastomotic leakage 4 days after operation; in the 5-FU1 group, one rat died from unknown causes 7 days after operation.

© Cancer Research Campaign 1999

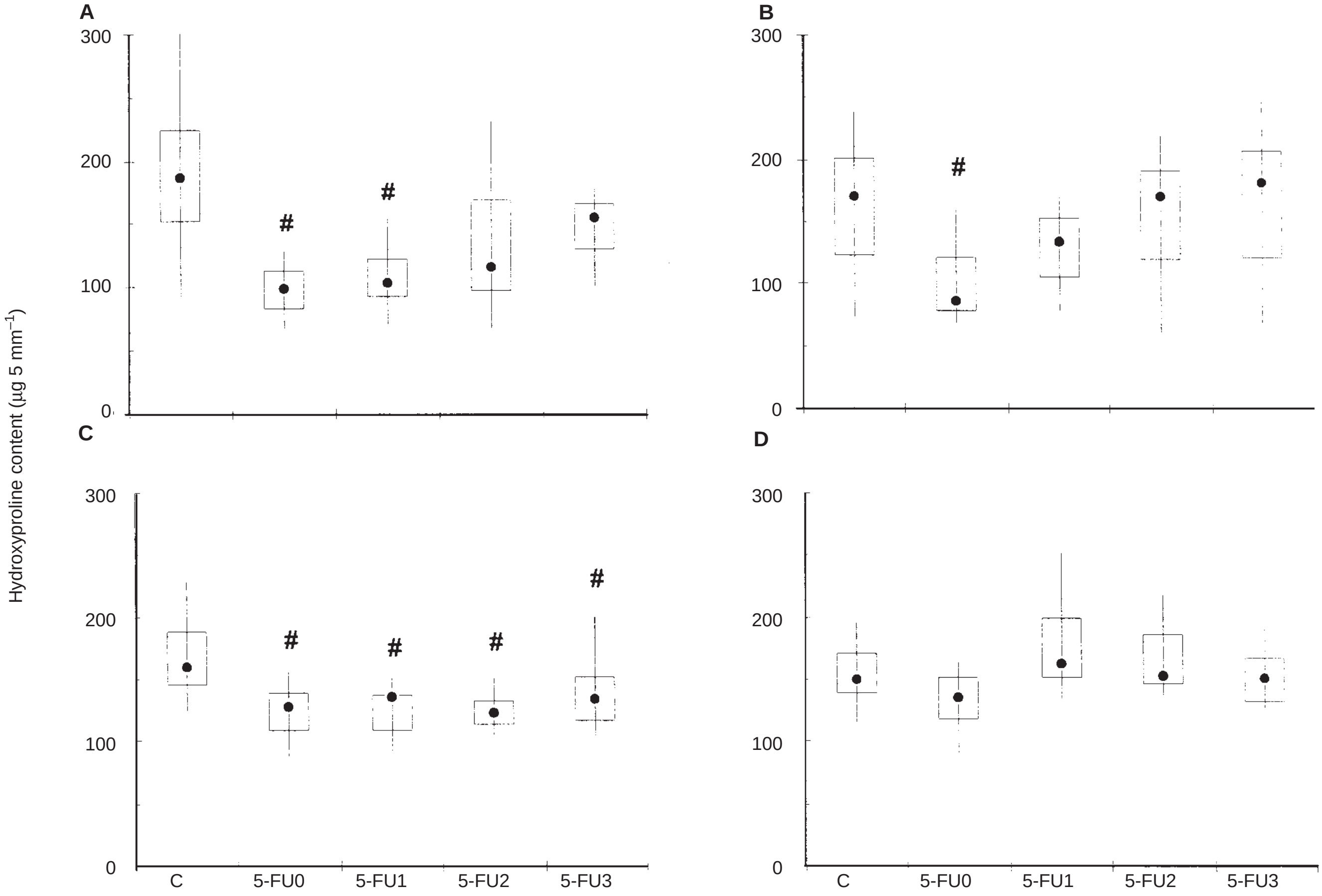

**Figure 3** Anastomotic hydroxyproline content. Data represent median values (●) and range (vertical lines); boxes delineate the 95% confidence interval. (**A**) Ileum, intraperitoneal 5-FU. (**B**) Ileum, intravenous 5-FU. (**C**) Colon, intraperitoneal 5-FU. (**D**) Colon, intravenous 5-FU. #Significant ($P < 0.025$, see Materials and methods) difference between experimental groups and the control group

At the end of the experiment, the average body weight in the control groups was equal to that immediately before operation. In all experimental groups, body weight was reduced. Average weight loss at day 7, calculated with respect to the weight before operation, was 28% in the 5-FU0 group, 24% in the 5-FU1 group, 17% in the 5-FU2 group and 13% in the 5-FU3 group after intraperitoneal drug administration. Corresponding values after intravenous 5-FU were 20, 19, 13 and 9% respectively. Statistical comparison (Mann–Whitney) between each experimental group and the control group yielded significance ($P < 0.0001$) in all cases.

Anastomotic strength was assessed in two ways: by the bursting pressure and, subsequently, by the breaking strength. Figure 1 depicts the bursting pressures measured in the individual anastomotic segments. Within the intraperitoneal group, the bursting pressure was strongly and significantly reduced in the 5-FU0, 5-FU1 and 5-FU2 groups, both in ileum (Figure 1A) and in colon (Figure 1C). Also, the bursting site, which was almost invariably outside the wound area in the control groups, had shifted significantly (Fisher's exact test) to the actual suture line. If 5-FU was started on the third day after operation (5-FU3), the bursting pressure in the anastomotic segment was equal to that of controls and significantly ($P < 0.0001$) higher than in each of the other 5-FU groups. However, in the ileum, wound strength was still reduced as indicated by the significant ($P = 0.0046$) shift in bursting site if compared with the controls. The loss of bursting pressure in the 5-FU groups seemed less severe if the drug was given intravenously. In the ileum (Figure 1B) the median values of both 5-FU2 and 5-FU3 groups were similar to that in the control group. However, loss of strength in the 5-FU2 group was still apparent from the significant ($P < 0.0001$, Fisher's exact test) shift of bursting site to the wound area. In the colon (Figure 1D), significant differences between the control and 5-FU groups were only apparent in the bursting site, which had shifted to the suture line in the 5-FU0 ($P < 0.0001$) and 5-FU1 ($P = 0.0006$) groups.

During the measurement of the breaking strength, tearing of the tissue always occurred within the suture line. The results are depicted in Figure 2. Intraperitoneal administration of 5-FU led to significant loss of ileal breaking strength (Figure 2A) in all experimental groups. Nevertheless, the reduction of strength was greatest (60%) in the 5-FU0 group and significantly ($P < 0.0001$) less so in the 5-FU2 and 5-FU3 groups. In the colon (Figure 2C) only the 5-FU0 group displayed loss of strength. If 5-FU was given intravenously, ileal anastomoses were significantly weakened in all experimental groups (Figure 2B) and colonic anastomoses only in the 5-FU0 and 5-FU1 groups.

The hydroxyproline content in the 5-mm tissue, which contained the actual anastomosis, was assayed as a measure for the presence of collagen. Figure 3 shows that intraperitoneal

© Cancer Research Campaign 1999

administration of 5-FU led to a reduced anastomotic hydroxyproline content. In the colon (Figure 3C) there was a significant decrease, with respect to the controls, in all experimental groups. In the ileum, this was the case for the 5-FU0 and 5-FU1 groups. Median values in the 5-FU2 and 5-FU3 groups were also lower than that in the control group, although these differences just failed to reach statistical significance ($P = 0.0317$ and $P = 0.0336$ respectively; cf. Materials and methods section). The effect of intravenous 5-FU was far less pronounced. Here, the only significant difference was observed in the ileum between the control and 5-FU0 group (Figure 3B).

### DISCUSSION

Adjuvant therapy in colorectal cancer is routinely withheld until weeks after operation. However, there exists an excellent rationale to start therapy in the immediate post-operative period (Fisher et al, 1983; Goldie and Coldman, 1985; Harris and Mastrangelo, 1991). Although, so far, limited information exists to support timing decisions in human neoplasms, there exists a growing perception that treatment with anti-cancer agents should begin at the day of surgery or as soon as possible thereafter (Cunliffe and Sugarbaker, 1989; Harris and Mastrangelo, 1991; Fielding et al, 1992).

Accepting the hypothesis that perioperative administration of 5-FU could benefit (certain classes of) patients after resection of intestinal malignancies, it becomes imperative to delineate accurately its potential detrimental effects on the healing anastomosis. As the early phase of the repair sequence depends heavily on cellular proliferation, adverse effects may be expected as a result of administration of non-specific anti-neoplastic agents during this period. Therefore, we analysed anastomotic strength 1 week after operation in terms of both bursting pressure and breaking strength. The former parameter is probably more sensitive in detecting localized loss of strength; a very small hole in the intestinal wall will result in loss of bursting pressure but not necessarily in a reduction in breaking strength. Within the present experiment, the outcome of both measurements is quite similar. The exception, a significant reduction in colonic bursting pressure without a concomitant reduction in breaking strength in the 5-FU1 and 5-FU2 groups after intraperitoneal administration of 5-FU, emphasizes the necessity of evaluating both parameters in order to draw the correct conclusion regarding the safety of any treatment schedule.

In a recent study, Weiber et al (1994) compared colonic healing during intraperitoneal 5-FU treatment, starting either at the day of operation or on the third post-operative day. Their findings indicate that the colonic breaking strength is not impaired when intraperitoneal 5-FU treatment is started at the later time point. We find this also to be true if, in addition, the bursting pressure is taken as the parameter for strength and if 5-FU is given intravenously. However, it is not valid for the intestine as a whole, because the breaking strength of ileal anastomoses remains significantly reduced in the 5-FU3 groups (Figure 2A and B). Furthermore, our data clearly demonstrate that anti-neoplastic therapy with 5-FU, if started on the first or second day after operation, may compromise the development of strength in intestinal anastomoses.

Wound strength is mainly dependent on the presence of collagen – alterations in both existing collagen, which is needed to anchor the sutures into the submucosa, and newly synthesized collagen, which is necessary to restore structural integrity to the bowel wall, can affect anastomotic strength. In most of our experimental groups, loss of strength is indeed accompanied by a significantly reduced hydroxyproline level in the anastomotic segment. Previous results, both in our animal model (de Waard et al, 1995*b*) and in human subcutaneous grafts (Graf et al, 1994*b*), have demonstrated that early post-operative intraperitoneal 5-FU treatment may strongly inhibit collagen synthetic capacity, possibly by a direct effect on fibroblast proliferation and collagen synthesis (de Waard et al, 1998). As no direct effects of 5-FU on collagen degradation are known, we suggest that reduced synthesis is primarily responsible for the observed loss of anastomotic strength. Although all experimental groups lose significantly more weight than the control groups, it seems unlikely that the (metabolic) effects of weight loss cause the reduction in wound strength, because nutritional depletion alone leading to weight loss of a similar magnitude does not lower anastomotic strength (Graf et al, 1994*a*).

The effects of intraperitoneal and intravenous 5-FU on anastomotic strength and hydroxyproline content appear qualitatively similar. However, local administration of the drug seems on the whole to result in a quantitatively more explicit response. For instance, after intraperitoneal administration the median bursting pressure in ileal anastomoses is reduced by 79% in the 5-FU0 group and by 55% in the 5-FU2 group; if delivered intravenously, the median reduction in these groups is 14% and 10% respectively. This agrees well with earlier data, which show that intraperitoneal administration of anti-neoplastic agents leads to a greater reduction of anastomotic collagen synthetic capacity than does intravenous delivery (Martens et al, 1992).

In conclusion, it can be stated that, within this animal model, post-operative systemic or intraperitoneal treatment with 5-FU, started before the third day after surgery, leads to loss of anastomotic strength. It remains to be seen what this means for clinical practice. As yet, few data are available, although trials to assess the efficacy of immediate post-operative systemic and regional chemotherapy are under way (Påhlman, 1995; Vaughn and Haller, 1997). It is quite possible that, for the individual patient, a period of suboptimal wound strength does not necessarily lead to anastomotic complications, as long as the strength reaches a certain minimal – so far undefined – value that ensures anastomotic integrity. However, clinicians should be well aware of the potential effects of early anti-neoplastic therapy, the more so if patients are treated presenting with conditions that may constitute additional threats to wound integrity. These may include, for example, malnutrition, bowel obstruction, peritonitis, (poorly controlled) diabetes, advanced age and, possibly, others (Golub et al, 1997; Thornton and Barbul, 1997). In these cases, the burden of two or more negative factors could be such that the risk for anastomotic leakage becomes unacceptable and postponement of adjuvant therapy is indicated.

### REFERENCES

Cunliffe WJ and Sugarbaker PH (1989) Gastrointestinal malignancy: rationale for adjuvant therapy using early postoperative intraperitoneal chemotherapy. *Br J Surg* **76**: 1082–1090

de Waard JWD, Wobbes T and Hendriks T (1993) Early post-operative 5-fluorouracil does not affect the healing of experimental intestinal anastomoses. *Int J Colorect Dis* **8**: 175–178

de Waard JWD, Wobbes T, de Man BM, van der Linden CJ and Hendriks T (1995*a*) Postoperative levamisole may compromise early healing of experimental intestinal anastomoses. *Br J Cancer* **72**: 456–460

de Waard JWD, Wobbes T, van der Linden CJ and Hendriks T (1995*b*) Vitamin A may promote 5-fluorouracil-suppressed healing of experimental intestinal anastomoses. *Arch Surg* **130**: 959–965

© Cancer Research Campaign 1999 

de Waard JWD, de Man BM, Wobbes T, van der Linden CJ and Hendriks T (1998) Inhibition of fibroblast collagen synthesis and proliferation by levamisole and 5-fluorouracil. *Eur J Cancer* **34**: 162–167

Fielding LP, Hittinger R, Grace RH and Fry JS (1992) Randomised controlled trial of adjuvant chemotherapy by portal-vein perfusion after curative resection for colorectal adenocarcinoma. *Lancet* **340**: 502–506

Fisher B, Gunduz N and Saffer E (1983) Influence of the interval between primary tumor removal and chemotherapy on kinetics and growth of metastases. *Cancer Res* **43**: 1488–1492

Fuchs CS and Mayer RJ (1995) Adjuvant chemotherapy for colon and rectal cancer. *Semin Oncol* **22**: 472–487

Goldie JH and Coldman AJ (1985) Genetic instability in the development of drug resistance. *Semin Oncol* **12**: 222–230

Golub R, Golub RW, Cantu R Jr and Stein DH (1997) A multivariate analysis of factors contributing to leakage of intestinal anastomoses. *J Am Coll Surg* **184**: 364–372

Graf W, Weiber S, Glimelius B, Jiborn H, Påhlman L and Zederfeldt B (1992) Influence of 5-fluorouracil and folinic acid on colonic healing: an experimental study in the rat. *Br J Surg* **79**: 825–828

Graf W, Weiber S, Jiborn H, Påhlman L, Glimelius B and Zederfeldt B (1994*a*) The roles of nutritional depletion and drug concentration in 5-fluorouracil-induced inhibition of colonic healing. *J Surg Res* **56**: 452–456

Graf W, Ivarsson M, Gerdin B, Hellsing K, Påhlman L and Glimelius B (1994*b*) The influence of early postoperative chemotherapy on human wound healing. *J Surg Res* **57**: 394–400

Harris DT and Mastrangelo MJ (1991) Theory and application of early systemic therapy. *Semin Oncol* **18**: 493–503

Hendriks T and Mastboom WJB (1990) Healing of experimental intestinal anastomoses: parameters for repair. *Dis Colon Rectum* **33**: 891–901

Martens MFWC, Hendriks T, Wobbes T and de Pont JJHHM (1992) Intraperitoneal cytostatics impair early post-operative collagen synthesis in experimental intestinal anastomoses. *Br J Cancer* **65**: 649–654

Påhlman L (1995) Open trials in colorectal cancer. *Eur J Surg Oncol* **21**: 347–351

Rustum YM, Harstrick A, Cao S, Vanhoefer U, Yin MB, Wilke H and Seeber S (1997). Thymidylate synthase inhibitors in cancer therapy: direct and indirect inhibitors. *J Clin Oncol* **15**: 389–400

Saltz LB and Kelsen DP (1997) Adjuvant treatment of colorectal cancer. *Annu Rev Med* **48**: 191–202

Sinicrope FA and Sugarman SM (1995) Role of adjuvant therapy in surgically resected colorectal carcinoma. *Gastroenterology* **109**: 984–993

Thornton FJ and Barbul A (1997) Healing in the gastrointestinal tract. *Surg Clin N Am* **77**: 549–573

Vaughn DJ and Haller DG (1997) Adjuvant therapy for colorectal cancer: past accomplishments, future directions. *Cancer Invest* **15**: 435–447

Weiber S, Graf W, Glimelius B, Jiborn H, Påhlman L and Zederfeldt B (1994) Experimental colonic healing in relation to timing of 5-fluorouracil therapy. *Br J Surg* **81**: 1677–1680

 *© Cancer Research Campaign 1999*
